# Outcome of testicular non-seminomatous germ cell tumours: report from a tertiary cancer centre in eastern India

**DOI:** 10.3332/ecancer.2021.1204

**Published:** 2021-03-11

**Authors:** Bivas Biswas, Deepak Dabkara, Sandip Ganguly, Joydeep Ghosh, Sujoy Gupta, Saugata Sen, Meheli Chatterjee, Archisman Basu, Satyadip Mukherjee

**Affiliations:** 1 Department of Medical Oncology, Tata Medical Center, 14 MAR (EW), New Town, Rajarhat, Kolkata 700160, India; 2 Department of Urosurgery, Tata Medical Center, 14 MAR (EW), New Town, Rajarhat, Kolkata 700160, India; 3 Department of Radiology, Tata Medical Center, 14 MAR (EW), New Town, Rajarhat, Kolkata 700160, India

**Keywords:** non-seminomatous germ cell tumour, testis, India, testicular violation

## Abstract

Non-seminomatous germ cell tumour (NSGCT) is a rare but highly curable malignancy. The literature on the management and outcomes of NSGCT is scarce from India. Here, we report the demography and treatment outcomes of NSGCT treated at our centre. This is a retrospective analysis of testicular and retroperitoneal NSGCT patients treated from March 2011 to December 2019. Patients were staged appropriately with imaging, pre- and post-operative tumour marker. Patients received stage adjusted adjuvant treatment after high inguinal orchiectomy. Patients with advanced disease were risk stratified as per International Germ Cell Cancer Collaborative Group (IGCCCG) classification. A total of 100 patients were treated with a median age of 28 years (Range: 18–51). Primary site was testis in 92 and retroperitoneum in 8 patients. Testicular violation was present in 17 (18%) patients. The stage of the disease was I in 32, II in 19 and III in 49 patients, respectively. IGCCCG risk groups were good in 29 (46%), intermediate in 13 (21%) and poor in 21 (33%) patients. Eleven patients (24%) underwent retroperitoneal lymph node dissection amongst 45 with post-chemotherapy residual disease. After a median follow-up of 26.6 months (range: 2.2–100.7), 3-year event-free survival and overall survival (OS) were 70.7% ± 5.6% and 78.2% ± 5.4%, respectively. S3 tumour marker (*p* = 0.01) and non-pulmonary visceral metastasis (*p* < 0.001) emerged as independent poor prognostic factors for OS in multivariate analysis. To conclude, testicular NSGCT has very high cure rate. Two-third patients present with advanced disease and one-third of them had poor risk disease. S3 tumour marker and non-pulmonary visceral metastasis are poor risk factors for OS.

## Introduction

Testicular germ cell tumours (TGCTs) constitute 1% of all adult malignancies [1, 2] and are the most common solid tumours in between 18 and 45 years of age [1, 3]. Incidence is lowest in African and Asian countries and highest in Scandinavian countries. Incidence of TGCT is increasing in Europe and the United States [2, 4, 5] and becoming more common in low- and middle-income countries [6]. India has one of the lowest incidences of TGCTs of 0.5 per 1 lakh men [7]. TGCT comprises seminomatous and non-seminomatous tumours and the latter is more aggressive.

There is a scarcity of literature on non-seminomatous germ cell tumours (NSGCTs) of testis from the Indian subcontinent with reports showing advanced stage of disease at presentation, scrotal violation and poor compliance to treatment [8–14]. Most of those publications are of very small patient numbers except the two latest publications [13, 14]. Testicular tumour has excellent cure rates and thus the focus should be on minimising the acute and long-term toxicities [15].

This study aims to analyse the clinicopathologic features, prognostic factors, treatment outcome and treatment-related toxicities in NSGCT evaluated and treated at our centre with research questions of – whether surgical aspects improved over time? and did our patients do well in terms of outcome as compared to published real-world literature.

## Materials and methods

### Patients

This is a retrospective study of male patients aged ≥ 18 years with testicular NSGCT evaluated and treated at our centre. Our centre is a non-profit state-of-the-art tertiary cancer care centre in Eastern India. We have different disease management groups for different area of cancer care in patients with solid tumours. The urooncology disease management group consists of four well qualified urosurgeons, two medical oncologists, two radiation oncologists along with a radiologist, pathologist and clinical nutritionist and manages a high number of all urological malignancies including approximately 30–40 cases of TGCT per year. We have also included a few patients with retroperitoneal primary as they are staged and treated like testicular primary. Patients were searched from a prospective database and hospital management services from March 2011 to December 2019. Patients who did not take any further treatment after initial staging and evaluation were excluded from this analysis. Ethical clearance waiver was obtained from the Institute Review Board as per institutional policy in view of the retrospective nature of this study. Patients with mediastinal primary were excluded from this analysis due to their different staging and treatment pattern. Clinicopathologic features, treatment pattern and outcome were recoded for data analysis.

### Diagnosis and staging

All eligible patients underwent testing for routine biochemical blood parameters, baseline tumour markers – alpha-fetoprotein (AFP), beta human chorionic gonadotrophin, lactate dehydrogenase, testicular ultrasound, computed tomography (CT) of chest and whole abdomen, semen analysis and sperm banking if family not completed and post-orchiectomy tumour markers. Pre-treatment tumour markers were considered for risk staging in case of retroperitoneal primary. Patients with seminoma histology but with elevated AFP or histological diagnosis of NSGCT or mixed GCT were treated as NSGCT.

Patients who presented to us after orchiectomy at another centre also underwent similar diagnostic testing along with pathology review of tumour blocks, whenever available. Bone scan was performed if clinically indicated or with high serum alkaline phosphatase. For advanced stage disease (stage II and stage III), patient had risk stratification as per International Germ Cell Cancer Collaborative Group (IGCCCG) classification [16].

### Treatment protocol, response evaluation and follow-up

Treatment decisions of all cases were decided after discussion by the multidisciplinary tumour board. Patients with stage I disease with adverse prognostic features (lymphovascular invasion, invasion of the spermatic cord or invasion of the scrotum) received two cycles of bleomycin, etoposide and cisplatin (BEP). Patients with metastatic disease were treated with BEP or etoposide/cisplatin (EP) or etoposide, ifosfamide and cisplatin (VIP) depending upon the IGCCCG risk stratification.

Tumour markers were re-checked before each cycles of chemotherapy in patients with advanced disease and elevated tumour markers. Response assessment was done by Response Evaluation Criteria in Solid Tumours v 1.1 [17] wherever applicable after preplanned chemotherapy cycles. Post chemotherapy resection of residual disease (retroperitoneal lymph node dissection (RPLND)) was done in case of normal tumour marker with ≥ 1 cm of residual disease after discussion in multidisciplinary tumour board and/or metastasectomy of residual metastatic site, wherever applicable and feasible. Patients were followed up 2–3 monthly for the first year, every 3–4 months for second and third year, every 6 months for fourth and fifth year and thereafter yearly after completion of treatment. Clinical examination and tumour markers were checked at each follow-up till third year and only clinical examination afterwards. CT scan of chest and whole abdomen was done during first year of follow-up and was replaced with ultrasound of whole abdomen and chest X-ray thereafter if no rise in tumour marker. Patients were followed up for acute and long-term treatment related toxicities as well as for recurrence. Patients who had weight gain on follow-up were also evaluated for signs and symptoms of metabolic syndrome including laboratory parameters as per American Heart Association/National Heart, Lung and Blood Institute Scientific Statement [18]. Those who has full component or partial component of metabolic syndrome were subjected to appropriate intervention [19] – weight reduction, regular exercise, life style modification and use of statin whenever indicated and were further followed-up for response to intervention.

### Statistical analysis

Descriptive statistics were used for demographics and clinical characteristics. Chi-square test was used to detect association between categorical variables. Student’s *t*-test was applied to compare continuous variables between groups. Survival was estimated by the Kaplan–Meier method and compared using log-rank test. Data were censored on 31 March 2020. The Cox proportional hazard model was used in univariate analysis to detect outcome differences between groups. Stepwise multivariate Cox regression analysis was done to identify the predictors of outcome. Factors with significance (*p* ≤ 0.1) in the univariate analysis were entered into multivariate analysis. Event-free-survival (EFS) with standard error was calculated from date of orchiectomy or date of diagnosis (in case of retroperitoneal primary) to date of disease relapse, disease progression or death due to any cause. Overall survival (OS) with standard error was calculated from date of diagnosis to date of death from any cause. Patients who were lost to follow-up or had treatment abandonment were also included for EFS and OS analysis and outcome in these patients was confirmed by telephonic contact. Treatment abandonment was included for survival analysis in the present study as it has been proposed that non-compliant and treatment abandonment patients should be included in survival analysis for studies from developing nations to provide a true picture of outcome from these countries [20]. STATA/SE 11.0 (StataCorp LP, Texas) was used for statistical analysis.

## Results

### Baseline characteristics

A total of 129 patients with a diagnosis of NSGCT were registered between March 2011 and December 2019, out of which 21 did not take any treatment (who either came for only medical opinion or opted for treatment elsewhere) and 8 had mediastinal primary, and hence were excluded from this analysis. Thus, 100 patients received treatment with a median age of 28 years (Range: 18–51). Clinicopathologic characteristics are mentioned in Table 1. Primary site of tumour was – testis in 92 and retroperitoneum in 8 patients. Testicular violation in the form of pre-operative fine needle aspiration or biopsy before presentation to our centre was present in 17 (18%, *n* = 92) patients.

### Treatment details and response

Stage of disease as per American Joint Committee on Cancer (AJCC) 7 was as follows – stage I in 32, stage II in 19 and stage III in 49 patients. Of evaluable 63 out of 68 advanced stage patients, the IGCCCG risk stratification was as follows – good risk in 29 (46%), intermediate risk in 13 (21%) and poor risk in 21 (33%) patients. Site of distant metastasis was lung in 32, liver in 7, bone in 4 and distant lymph nodes in 20 patients. High inguinal orchiectomy was performed in 78 (86%, *n* = 92) patients at our centre and 14 (14%) patients had scrotal orchietomy before presenting to us. Pre-orchiectomy tumour markers were available in 68% (*n* =63/92) patients. Histological reports of different component of germ cell in orchiectomy specimen are mentioned in Table 2.

Patients with stage I NSGCT (*n* = 32) after orchiectomy received treatment as follows – surveillance in 17, prophylactic RPLND in 1, BEP in 12 and EP in 2 patients. Patients with stage II NSGCT (*n* = 19) received treatment as follows – upfront RPLND in 2, BEP in 9 and EP in 8 patients. Chemotherapy details in stage III patients (*n* = 49) were – BEP in 34, EP in 13 and VIP in 2 patients. Response to first line chemotherapy in advanced stage disease (*n* = 68) was complete response in 19 (28%), partial response in 42 (62%), stable disease in 3 (4%) and progressive disease in 4 (6%) patients.

### Toxicity

From the available recodes of 80 patients, 28 (35%) patients had any grade toxicity as follows: bleomycin induced lung injury – 4 (grade 2 in 2 and grade 3 in 2), febrile neutropenia (FN) – 14 (grade 2 in 2 and grade 3 in 12), vomiting – 3 (grade 2 in 1 and grade 3 in 2), loose motion – 3 (grade 1 in 2 and grade 2 in 1), symptomatic hyponatraemia – 1 (grade 3), psychosis – 1 (grade 2) and myocardial infarction – 1 (grade 3). Eighteen patients required hospitalisation due to grade 3 or 4 nature of toxicity without any toxicity related mortality.

### Metabolic syndrome

Twenty-seven (47%) out of evaluable 58 patients had features of metabolic syndrome during follow-up that include excessive and unexpected weight gain, deranged fasting lipid profile (high LDL cholesterol, low HDL, high triglyceride), high blood sugar and increase in blood pressure.

### Management of residual disease

In patients with advanced disease (*n* = 68), 45 patients had residual disease after initial chemotherapy. Thirteen patients (29%) underwent RPLND. None had metastasectomy for residual metastatic disease. Viable tumour detected in six, mature teratoma and necrosis in three, complete tumour necrosis in two and only mature teratoma in two patients.

### Salvage therapy

Twenty-four patients had relapse or progression in disease. Site of progression was – thorax (lung, thoracic lymph nodes or pleura) in 8, progressively increasing markers in 8, retroperitoneal lymph nodes in 6, brain in 1, bone in 1 patient and 1 patient had sarcomatoid transformation. Four (13%, *n* = 4/32) patients in stage I, 3 (16%, *n* = 3/19) patients in stage II and 17 (35%, *n* = 17/49) patients in stage III had progressive/recurrent disease later on. Fifteen (63%) patients received second line treatment and chemotherapy regimens were VIP in 10, EP in 1, paclitaxel/ifosfamide/platinum in 3 and vinblastine/ifosfamide/platinum in 1 patient. Response to second line treatment was – complete response in 2, partial response in 6, stable disease in 1 and progressive disease in 6 patients. Three patients underwent third line treatment. None of the patients underwent/opted for high dose chemotherapy followed-by stem cell rescue.

### Survival analysis

**Figure 1.**
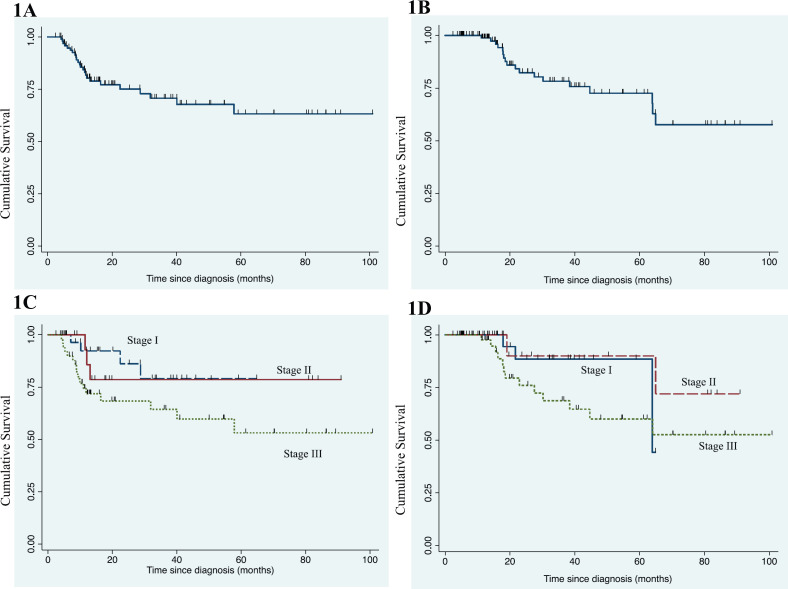
(a): Kaplan-–Meier graph for EFS in entire cohort. (b): Kaplan–Meier graph for OS in entire cohort. (c): Kaplan–Meier graph for EFS according to stage in advanced stage disease. (d): Kaplan–Meier graph for OS according to stage in advanced stage disease.

After a median follow-up of 26.6 months (95% confidence interval (CI): 19.6–38, range: 2.2–100.7), median EFS was not reached. The 3-year EFS and OS were 70.7% ± 5.6% and 78.2% ± 5.4% (Figure 1a and b), respectively. The 3-year EFS was 79% ± 9.9%, 78.6% ± 11% and 64.4% ± 8.5% and 3-year OS was 88.5% ± 7.6%, 90% ± 9.5% and 68.8% ± 8.3% in stage I, stage II and stage III, respectively (Figure 1c and d). Eighteen patients died till data cut-off and survival status is not known in 25 patients who were lost to follow-up subsequently.

### Univariate and multivariate analysis

**Figure 2.**
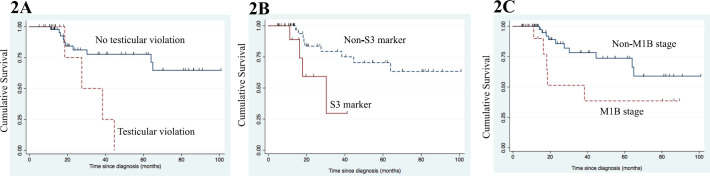
(a): Kaplan–Meier graph for OS according to testicular violation in advanced stage disease. (b): Kaplan–Meier graph for OS according to S3 marker status in advanced stage disease. (c): Kaplan–Meier graph for OS according to non-pulmonary visceral metastasis in advanced stage disease.

None of the clinical factors revealed significance for EFS in univariate analysis (Table 3). Age (*p* = 0.06), IGCCCG risk group (*p* = 0.06), types of chemotherapy regimen (*p* = 0.07), non-pulmonary visceral metastasis (*p* = 0.09) also did not show significance for EFS estimate in univariate and multivariate analysis. Testicular violation (*p* = 0.02) (Figure 2a) and S3 tumour maker (*p* = 0.04) (Figure 2b) predicted inferior OS in univariate analysis (Table 3). S3 tumour marker (hazard ratio (HR): 6.15; 95% CI: 1.44–26.2, *p* = 0.01) and non-pulmonary visceral metastasis (HR: 15.9; 95% CI: 3.95–64.5, *p* < 0.001) (Figure 2c) emerged as independent poor prognostic factors for OS in multivariate analysis.

## Discussion

Testicular GCT is a highly curable malignancy which predominantly affects adolescents and young adults. Testicular cancers are rare in India with incidence of 0.5–1 per lakh population [1]. This is real world data of NSGCT patients treated in a tertiary care cancer centre in India. Many patients are not referred or are referred late to tertiary care hospitals from the community physicians or surgeons. Most of the patients who were referred at our centre are had prior orchiectomy (and/or testicular biopsy), do not had preoperative tumour markers or no proper risk stratifications were available which sometimes make treatment decisions difficult. The results of our study reflect those findings.

Well established data are available from developed countries with mature outcome data. Apart from two with large amounts of data [13, 14], there are only a few published reports available from India on NSGCT [8–12]. Those are also limited by improper risk stratification or treatment details and minimal outcome data (Table 4). The majority of patients from India present with a heavy burden of disease, with bulky retroperitoneal lymph nodes [13, 14]. Similar results are reflected in our publication as one-third of patients presented with poor risk disease (Table 1). These factors along with poor treatment compliance and treatment abandonment lead to poor survival in patients in developing countries as compared to developed countries.

Scrotal violation in the form of testicular biopsy or scrotal orchiectomy is a well-documented poor risk factor for OS as mentioned in literature [13, 21, 22] owing to disruption of lymphatic drainage and resultant increased local recurrence. Seventeen (18%) patients in our cohort had testicular violation (testicular biopsy – 3 and scrotal orchiectomy – 14) before presenting to us. Lack of awareness about the disease entity or prognostic factors and treatment of NSGCT amongst primary care physician or community surgeons leads to this phenomenon in India and almost reflected in each of the publications. Creating awareness about this highly curable disease among primary care physicians or surgeons is the key to successful outcomes for NSGCT in India. IGCCCG poor risk group was identified as a poor risk factor in previous studies including a study from India [13] which reflects the burden of disease and similarly, in our series S3 marker emerged as a poor prognostic factor for OS. Another study from India [14] reported non-pulmonary visceral metastasis as a poor prognostic factor for outcome similar to our finding (Figure 2c).

The role of bleomycin in the treatment of NSGCT has been questioned recently in view of its contribution to disease control compared to high pulmonary toxicity and post-op mortality [23, 24]. Alternative regimen has been studied in poor risk NSGCT which gives comparable efficacy but less toxicity and VIP regimen is the current better alternative for that especially in those with mediastinal primary [25]. Our trend in recent times also shifted towards the use of VIP regimen replacing BEP in poor risk NSGCT. A few of our patients also developed bleomycin induced pulmonary toxicity (Table 4). Patients who received BEP showed a trend towards poor outcome in our cohort. This is expected as the majority of patients with poor risk disease received BEP regimens.

None of the previous Indian studies reported the components of GCT in biopsied or resected specimens. Our study has reported the various components of GCT and/or mature teratoma in orchiectomy specimen or resected residual disease (Table 2). One-fourth (*N* = 23/92) of patients had mixed components of seminoma and NSGCT and 2 patients had somatic differentiation. The strength of our study was that all patients were treated after discussion in the multidisciplinary tumour board and all patient related information was recorded prospectively in an electronic medical database. All patients were followed-up closely and salvaged appropriately on early detection of recurrence. Patients were monitored for treatment related to long-term complications like – metabolic syndrome and timely intervention occurred.

Our study has many limitations which may affect the clinical interpretation of the study results. It was retrospective in nature with inherent missing data. Many patients were referred after orchiectomy and partial treatment without risk stratification. Treatment abandonment, lost to follow-up and inadequate treatment are key factors for poor oncological outcome of NSGCT in developing nations. Many eligible patients defaulted for RPLND. Forty-two patients defaulted for further treatment either due to progressive disease or many other unknown reasons which include logistics and finance. The survival status of those defaulted patients is not known.

## Conclusions

Testicular NSGCT is a rare malignancy with high cure rates. Patients should be treated in a tertiary care centre with multidisciplinary management. S3 tumour marker and non-pulmonary visceral metastasis are poor prognostic factors for survival. Education of primary care physicians and general surgeons is the key to avoid testicular violation and immediate referral to tertiary care cancer centres. In view of the high cure rate, patient should be followed up for a long time for early detection of treatment related complications, such as metabolic syndrome and second malignancies. Our study is a real world picture of management, patient compliance and outcome of treatment in a resource limited setting.

## Funding

None.

## Conflicts of interest

The author(s) declare that they have no conflict of interest.

## Authors contribution

All authors contributed to idea development, methodology, data acquisition, data analysis and interpretation, manuscript writing and editing.

## Figures and Tables

**Table 1.** Clinicopathologic features.

Variables	Number	%
Age (years)	28 (median)	18–51 (range)
Symptom duration (months)	4 (median)	0.3–72 (range)
**Site of primary**		
Testis	92	92
Retroperitoneum	08	08
**Cryptorchism** (*n* = 92)		
Yes	02	02
No	90	98
**Testicular violation** (*n* = 92)		
Yes	17	18
No	75	82
**Orchiectomy type** (*n* = 92)		
High inguinal	78	85
Scrotal	14	15
**Semen analysis**		
Yes	20	20
No	80	80
**Tumour marker^a^** (*n* = 89)		
S0	29	33
S1	27	30
S2	22	25
S3	11	12
**Stage of the disease**		
I	32	32
II	19	19
III	49	49
**IGCCCG risk group^b^** (*n* = 63)		
Good	29	46
Intermediate	13	21
Poor	21	33

a Post-orchiectomy in testicular primary (data not available in three patients)

b Risk stratification not available in five patients

IGCCCG, International Germ Cell Cancer Collaborative Group

**Table 2.** Histological details (*n* = 92).

Types of components	Number
Embryonal carcinoma	10
Embryonal carcinoma + immature teratoma	1
Embryonal carcinoma + yolk sac tumour	7
Embryonal carcinoma + yolk sac tumour + immature teratoma	1
Embryonal carcinoma + yolk sac tumour + choriocarcinoma	2
Embryonal carcinoma + yolk sac tumour + teratoma	7
Embryonal carcinoma + teratoma	4
Non-seminomatous germ cell component, unclassified	6
Seminoma + embryonal carcinoma	8
Seminoma + embryonal carcinoma + choriocarcinoma	1
Seminoma + embryonal carcinoma + immature teratoma	1
Seminoma + immature teratoma	2
Seminoma + yolk sac tumour	4
Seminoma + yolk sac tumour + immature teratoma	2
Seminoma + yolk sac tumour + teratoma	2
Seminoma + yolk sac tumour + teratoma + embryonal carcinoma	1
Seminoma + teratoma	2
Yolk sac tumour	6
Yolk sac tumour + immature teratoma	4
Yolk sac tumour + immature teratoma + choriocarcinoma	1
Yolk sac tumour + teratoma	5
Yolk sac tumour + teratoma + choriocarcinoma	1
Mixed germ cell component, unclassified	8
Immature teratoma	1
Teratoma	3
Teratoma + somatic differentiation	2

**Table 3.** Univariate analysis for EFS and OS in advanced disease (stages II and III, *n* = 68).

		EFS			OS		
Variables	Number	HR	95% CI	*p*	HR	95% CI	*p*
							
Site of primary	Testicular (*n* = 60)	1		0.94	1		0.59
	Retroperitoneum (*n* = 8)	2.03	0.46–8.94		1.74	0.22–13.45	
							
Age	≤28 years (*n* = 35)	1		0.06	1		0.07
	>28 years (*n* = 33)	0.39	0.15–1.03		0.35	0.11–1.09	
							
Symptom duration^a^	≤4 months (*n* = 27)	1		0.63	1		0.7
	>4 months (*n* = 24)	0.76	0.25–2.28		1.25	0.38–4.13	
							
Testicular violation	No (*n* = 49)	1		0.39	1		0.02
	Yes (*n* = 11)	1.66	0.57–5.21		4.24	1.26–14.27	
							
Type of orchiectomy^b^	High inguinal (*n* = 50)	1		0.24	1		0.32
	Scrotal (*n* = 9)	1.96	0.64–6.02		1.92	0.53–6.98	
							
Stage of disease	Stage II (*n* = 19)	1		0.19	1		0.21
	Stage III (*n* = 49)	2.26	0.66–7.34		2.59	0.58–11.55	
							
IGCCCG risk group^c^	Good (*n* = 29)	1			1		
	Intermediate (*n* = 13)	3.09	0.98–9.75	0.06	1.83	0.45–7.39	0.4
	Poor (*n* = 21)	2.64	0.83–8.36	0.1	2.87	0.81–10.3	0.1
							
S3 marker (*n* = 60)	No (*n* = 49)	1		0.18	1		0.04
	Yes (*n* = 11)	2.20	0.70–6.92		3.74	1.07–13.04	
							
Non-pulmonary visceral mets	Absent (*n* = 55)	1		0.09	1		0.08
	Present (*n* = 13)	2.32	0.89–6.07		2.59	0.88–7.64	
							
Chemotherapy type^d^	BEP (*n* = 43)	1		0.07	1		0.07
	EP (*n* = 21)	0.32	0.09–1.08		0.15	0.02–1.28	

a Symptom duration not recorded in 17 patients

b One patient did not undergo orchiectomy

c Risk stratification not available in five patients

d Two patients received VIP based chemotherapy and another two patients underwent RPLND

BEP, Bleomycin/etoposide/cisplatin; CI, Confidence interval; EFS, Event free survival; EP, Etoposide/cisplatin; HR, Hazard ratio, IGCCCG, International Germ Cell Cancer Collaborative Group; OS, Overall survival

**Table 4.** Comparative study of Indian publication on NSGCT.

Study (year)	Number	Sites	Risk groups	Treatment	ORR	Toxicity	RPLND	EFS	OS
Raina *et al* [11]	63	Testis	NR	PBV + VAB-6: 100%	81%	NR	NR	NR	3 yr – 80%
Bhutani *et al* [8]	53	NR	Good – 41% Intermediate – 17% Poor – 40%	NR	92%^a^	Bleomycin lung injury – 1 (grade 5) FN – 4 (grade 5)	12%	2 yr – 57%	2 yr – 70%
Singh *et al* [12]	48	Testis	Good risk – NR Intermediate risk – NR Poor risk – 40%	NR	NR	NR	NR	NR	NR
Joshi *et al* [10]	50	Testis	Good – 9% Intermediate – 53% Poor – 38%	BEP – 80% EP – 20%	78%	FN – 22%, haematological – 53%, non-haemat – 13%	40%	NR	NR
Saju *et al* [13]	293	Extra-cranial	Good – 32% Intermediate – 30% Poor – 38%	BEP – 78% EP – 14% Carboplatin – 2% Others – 6%	82%	^a^FN – 13%, ^a^Bleomycin lung injury – 4	41%	3 yr – 67.4%	3 yr – 75.3%
Nair *et al* [14]	119	Testis	Good – 52% Intermediate – 28% Poor – 20%	All BEP (except EP – 1 and VIP – 1)	59% (CR)	FN – 12, bleomycin lung injury – 8	*N* = 14	4 yr – 84.5%	4 yr – 87.1%
Singh *et al* [9]	19	Testis	^a^Good – 66% ^a^Intermediate – 13% ^a^Poor – 21%	NR	79%^a^	FN – 5.7%, Diarrhoea – 11%	NR	3 yr – 69.2%	3 yr – 71.4%
Current study (2020)	100	Testis, RP	Good – 46% Intermediate – 21% Poor – 33%	BEP – 69% EP – 29% VIP – 2%	90%	FN – 18%, bleomycin lung injury – 4	24%	5 yr – 63.3%	5 yr – 72.6%

a Combined seminoma and NSGCT

BEP, Bleomycin/etoposide/cisplatin; CR, Complete remission; EFS, Event free survival; EP, Etoposide/cisplatin; FN, Febrile neutropenia; ORR, Overall response rate; NR, Not reported; OS, Overall survival; PBV, Cisplatin/vinorelbine/bleomycin; RPLND, Retroperitoneal lymph node dissection; VAB-6, Cisplatin/vinblastine/actinomycin-D/bleomycin/cyclophosphamide; RP, retroperitoneum
